# Automatic stent strut detection in intravascular optical coherence tomographic pullback runs

**DOI:** 10.1007/s10554-012-0064-y

**Published:** 2012-05-23

**Authors:** Ancong Wang, Jeroen Eggermont, Niels Dekker, Hector M. Garcia-Garcia, Ravindra Pawar, Johan H. C. Reiber, Jouke Dijkstra

**Affiliations:** 1 LKEB–Division of Image Processing, Department of Radiology, Leiden University Medical Center, P. O. Box 9600, Leiden, Netherlands; 2 Cardialysis BV, P. O. Box 2125, Rotterdam, Netherlands

**Keywords:** IVOCT, Stent analysis, Strut detection, Strut segmentation, Guide wire removal

## Abstract

We developed and evaluated an automatic stent strut detection method in intravascular optical coherence tomography (IVOCT) pullback runs. Providing very high resolution images, IVOCT has been rapidly accepted as a coronary imaging modality for the optimization of the stenting procedure and its follow-up evaluation based on stent strut analysis. However, given the large number of struts visible in a pullback run, quantitative three-dimensional analysis is only feasible when the strut detection is performed automatically. The presented method first detects the candidate pixels using both a global intensity histogram and the intensity profile of each A-line. Gaussian smoothing is applied followed by specified Prewitt compass filters to detect the trailing shadow of each strut. Next, the candidate pixels are clustered using the shadow information. In the final step, several filters are applied to remove the false positives such as the guide wire. Our new method requires neither a priori knowledge of the strut status nor the lumen/vessel contours. In total, 10 IVOCT pullback runs from a 1-year follow-up study were used for validation purposes. 18,311 struts were divided into three strut status categories (malapposition, apposition or covered) and classified based on the image quality (high, medium or low). The inter-observer agreement is 95 %. The sensitivity was defined as the ratio of the number of true positives and the total number of struts in the expert defined result. The proposed approach demonstrated an average sensitivity of 94 %. For malapposed, apposed and covered stent struts, the sensitivity of the method is respectively 91, 93 and 94 %, which shows the robustness towards different situations. The presented method can detect struts automatically regardless of the strut status or the image quality, and thus can be used for quantitative measurement, 3D reconstruction and visualization of the stents in IVOCT pullback runs.

## Introduction

Heart disease is a leading cause of death in the developed countries and coronary artery disease (CAD) is the most common form [1]. In the treatment of CAD, stents are placed in the coronary arteries by means of the percutaneous coronary intervention (PCI) procedure. A stent is a tiny tube-like structure that is usually made of a wire mesh which is designed to be inserted into a vessel and functions as a scaffold device to keep the vessel open. The first generation of stents were bare metal stents, which have proven to be associated with an increased risk of coronary restenosis during the vessel wall healing process based on long term follow up studies [2, 3]. The second generation—drug eluting stents (DES) significantly decreased the occurrence of restenosis, but they are associated with late acquired stent malapposition which may lead to in-stent thrombosis [4]. Although newly implanted stents usually are located at the lumen boundary without tissue coverage (apposition) and later on nicely covered with a thin layer of tissue, still acute malapposition may occur or they may obstruct the blood flow to side-branches [5]. Therefore, detecting the stent strut position is highly important for stent placement evaluation and its follow-up analysis.

**Fig. 1:**
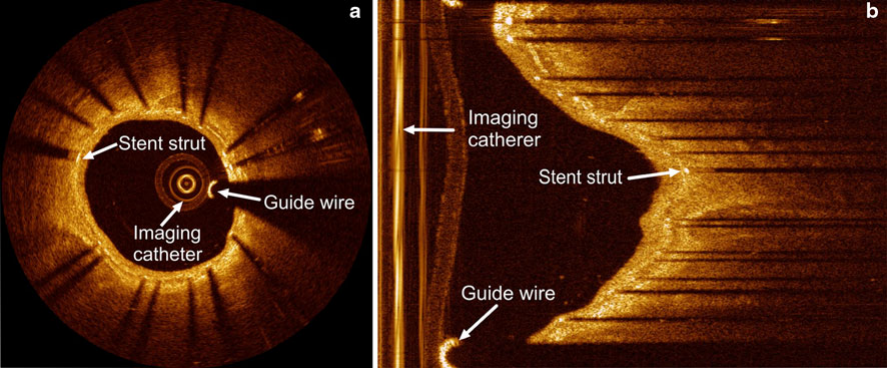
Examples of IVOCT images in **a** Cartesian coordinate system and in **b** polar coordinate system respectively. In both images, a stent strut, the guide wire and the imaging catheter are annotated

Intravascular ultrasound (IVUS) has been used for automatic stent strut detection, but its limited spatial resolution and low signal-to-noise ratio makes the detection difficult. To the best of our knowledge, no paper has been published for precise strut segmentation in IVUS pullback runs. As a relatively new optical signal acquisition technique, IVOCT imaging has a very high resolution (10–20 μm) which is about ten times higher than IVUS. IVOCT has been used as the exclusive technology for the precise in vivo evaluation of strut coverage and vessel wall healing [6–8]. The acquisition is performed similar to IVUS; the imaging catheter acquires cross-sectional images of the coronary artery by emitting near infrared (NIR) light instead of ultrasound towards the vessel wall in a radial manner while the transducer is rotating and the catheter is pulled back with a high and constant pullback speed. The superior sensitivity of the newly developed frequency-domain OCT systems (OFDI) is not only a key factor in achieving high image resolution, but also an important prerequisite for high speed imaging. It allows an acquisition speed of 100–160 frames per second and a very fast pull back speed (15–25 mm/s) which highly decreases the imaging time; on the other hand, it results into a large amount of images for each single procedure [9, 10]. Two IVOCT images in different coordinate systems are shown in Fig. 1.

Research is being carried out worldwide on IVOCT images, but an automated strut detection method that works robustly on routinely acquired clinical datasets remains a challenge. Many studies still depend on the manual strut detection. Two approaches [11, 12] require the lumen and vessel wall contour to define the region of interest (ROI), and subsequently detect the newly implanted and covered struts in two different modes. For severely malapposed struts, they may be located outside the ROI and therefore cannot be detected. Another approach [13] detects the strut luminal surface in each A-line (scan-lines in the polar image) using flexible intensity thresholds. A priori knowledge of the strut status (apposed or covered) is needed for stent strut detection. The type of the implanted device is also required for apposition assessment. The catheter artifacts and guide wire are masked with a fixed region and the guide wires beyond the mask region are manually detected and removed, which may be time-consuming.

Separating modes for different strut status: “malapposition”, “apposition” and “covered” usually can improve the detection accuracy, but a pullback run or even a single image may contain struts with different status. In this paper, we present a robust algorithm to process an entire IVOCT pullback run, which requires neither a priori status information, nor lumen or vessel wall contours.

## Materials

During this research, all of our IVOCT pullback runs were acquired using a C7-XR FD-OCT intravascular imaging system with a C7 Dragonfly™ Intravascular Imaging catheter (LightLab Imaging, Inc., Westford, MA, USA). The intravascular imaging catheter works together with a 6F guiding catheter. The automated pullback speed is 20 mm/s with a data frame rate of 100 frames per second. During the acquisition, a standard 0.014 inch steerable guide wire may be used. Temporary blood flushing is performed with a contrast infusion.

We use the 16-bit raw image data in polar coordinate system instead of the commonly used 8-bit Cartesian image representation [12], because it contains all of the original information and some details might get lost during the conversion from polar to Cartesian. Each polar frame has the same size of 960 × 504 pixels.

Although all the pullback runs were acquired with the same IVOCT system, they differ significantly in image quality. There are multiple reasons for that: for example, the noise can be caused by the residual blood after the infusion or by tiny air bubbles [7]. Moreover, the limited penetration depth, imaging catheter position, cardiac motion, redundant echo and many other factors can also affect the image quality.

## Method

### General approach for stent strut detection

**Fig. 2:**
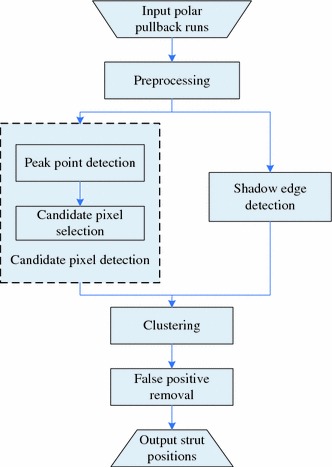
Flow chart of the strut detection algorithm processing steps

The automatic strut detection method was developed using the MeVisLab toolbox (MeVis Medical Solutions AG, Bremen, Germany) together with in-house developed C++ modules. As the flow chart in Fig. 2 shows, our detection method consists of five steps: first, the pullback runs are preprocessed to de-noise and define the proper ROI. Next, the strut candidate pixels are detected by locating the front edges of the struts in IVOCT images. In order to remove false candidate pixels and to cluster the remaining pixels into discrete struts, the shadow edges are detected. Finally after clustering, the guide wire and some false positives are removed using 3D information of the whole pullback run. In the following sections, each of the subsequent steps for the strut detection in IVOCT is described in further detail.

### Preprocessing

The preprocessing starts with noise reduction in the IVOCT pullback run since it hampers the strut detection. The main part of the noise has a relatively low intensity value. According to Ughi et al. [13], the lowest 5 % intensity values of the histogram can be considered as noise. In a similar fashion, we determine the histogram of the whole pullback run and set all pixels below this threshold value to 0.

The preprocessing continues with the definition of the Region of Interest (ROI). In order to detect also the malapposed struts, we need to select a bigger ROI than the region between lumen contour and vessel wall contour. However in the lumen area, the imaging catheter may generate very bright artifacts which have similar intensity values as stent struts. The ROI should exclude these artifacts.

The catheter artifact appears like rings in the center of the image as Fig. 1a shows. After a proper z-offset correction, they are constant in all frames of a single pullback run [14]. In the polar data, these artifacts are shown as parallel vertical lines at the left side of the images and they may affect the strut detection. To exclude these artifacts, a minimum filter in z-direction and a vertical line detection method [15] are applied to each IVOCT pullback run. The region to the right of these continuous straight vertical lines determines the ROI for our detection method.

### Candidate pixel detection

**Fig. 3:**
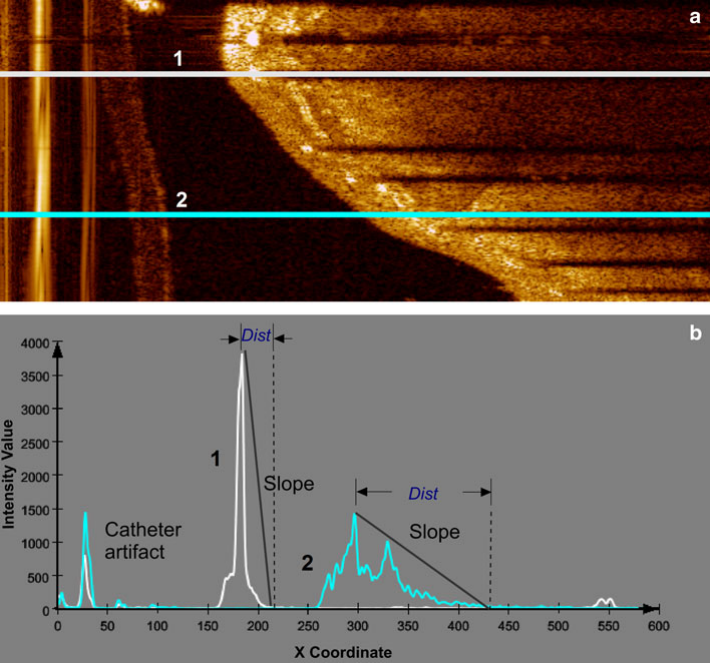
Examples of intensity profiles in polar images. The A-line (*1*) in **a** crosses a stent strut and its corresponding intensity profile in **b** has a higher peak point and a sharp fall to the *shadow area*, compared to the A-line (*2*) in **a**, which passes purely through tissue and its corresponding intensity profile in **b** has a longer distance between the peak point and the *shadow area*. “*Dist*” indicates the distance between the peak point and the start point of the trailing shadow

In IVOCT images, a metal stent strut appears normally as a bright spot with a trailing shadow behind it, since the strut reflects most of the light, while normal vessel tissue scatters and attenuates the light. Therefore, a strut has higher intensity values than the surrounding tissue. The pixels having the maximum intensity value in each A-line are candidates, under the assumption that there is only one strut per A-line. This also means that currently we exclude overlapping stents. Figure 3 shows two examples of the intensity profile.

**Fig. 4:**
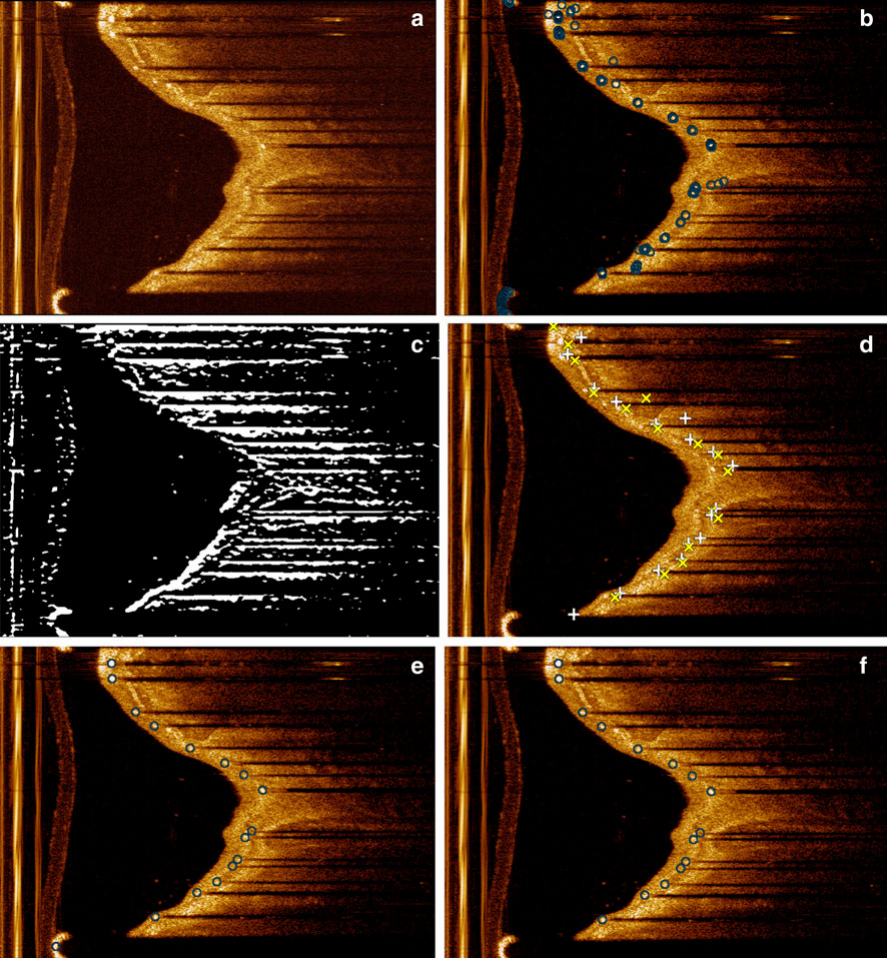
Results of each step in the shadow edge detection. **a** shows the original image. **b** Shows the results of the candidate pixel detection including false positives. Peak points are indicated as ‘*circle*’. **c** shows the result after Prewitt compass edge detection for only bottom edges. In **d**, the top edges are indicated by ‘*plus sign*’ and the bottom edges by ‘*times* *symbol*’. **e** Shows the clustering results which are indicated by ‘*circle*’. In **f** the final results after guide wire removal are presented with the struts indicated by ‘*circle*’

In general, one cannot state that the struts always have the highest intensity values in an entire pullback run. For that reason, a global intensity threshold is not applicable, and we have decided to use the slope of the intensity profile. By detecting the maximum intensity and the distance between this peak point and the first pixel of the potential shadow area, the slope is calculated. The potential shadow area is defined by a window of 30 continuous low intensity pixels. The maximum intensity value of the potential shadow region was set as the 89th percentile of the intensity histogram of the ROI in the entire pullback run. The slope reflects the local intensity change, and strut pixels usually are associated with a steeper slope than tissue pixels. An example of the candidate pixel results is shown in Fig. 4b. Because the distance from struts to their trailing shadows are similar, we determine the slope threshold based on the histogram as well.

### Shadow edge detection

To cope with artifacts such as the sunflower artifact [14], the position of a stent strut is defined by the middle point of its front edge. It reflects the start position of a strut, which can be used for the quantitative analysis of strut and the 3D stent reconstruction.

The middle point is calculated by the average position of a group of candidate pixels. However, it is difficult to cluster the candidate pixels into individual struts directly, because both the width of the struts and the gap between two neighboring struts can vary significantly. We also need to remove the false candidate pixels most of which are located in the tissue area outside the struts regions as Fig. 4b shows. In order to solve these issues, the width and the location of each strut are needed. Additional information is gathered by using the trailing shadows behind the struts. As the trailing shadows align with the imaging catheter, they are almost horizontal in the polar image, and their width and location are approximations of the corresponding strut width and location. The top and the bottom edge of a shadow define the clustering region for the strut.

A Gaussian filter is applied to smooth the images before the shadow edge detection. Next, a Prewitt compass operator with two special kernels is applied to detect the top and bottom edges separately [16]: one kernel is only sensitive to the horizontal bright to dark edges (top edges); while the other kernel is only sensitive to the horizontal dark to bright edges (bottom edges). An example of the bottom edges is shown in Fig. 4c. Only edges above a certain length were accepted, to avoid the short false shadow edges such as those associated with an eccentric lumen boundary.

### Clustering

The detected edges divide the polar images into consecutive intervals, which define the location and width of the struts. We cluster the candidate pixels in each interval. Special attention is paid to edges at the top and bottom of the polar images, since they actually could belong to each other, but have been split into two halves due to the nature of the polar image. At the start of the clustering, each candidate pixel is a cluster [17]. Clusters merge if the minimal distance between them is shorter than a threshold, in our case determined experimentally at a 4 pixel distance. This procedure continues until no more clusters can merge.

In some cases, only one edge of the shadow can be detected, because the other edge is too short or too blurred, especially when a strut is located far away from the imaging catheter. At the same time, false edges may be included if there is e.g. a seaming artifact [12] or highly eccentric lumen boundary. An example of the seaming artifact is shown in Fig. 6c. To avoid the false clusters caused by these influences, we need to select the correct candidate struts from the clusters. Because the strut is right below its top edge and above its bottom edge, for each top edge, the first cluster below it will be selected. Similarly, for each detected bottom edge, the first cluster above it will be selected. All the other clusters are removed. The average position of the candidate pixels of the same cluster determines the corresponding strut position.

In cases where the struts are located far away from the imaging catheter or covered with a thick layer of hyperplasia, a strut may have a low intensity value comparable to the surrounding tissue, and the described candidate pixel detection may fail. If there is no candidate strut between a pair of top and bottom edges, we check if there is a non-bright strut. We first assign a search range based on the start points of the shadow edges. In this search range, the pixel with the highest intensity value of each A-line in the shadow region is detected. All the pixels with an intensity value higher than the maximum shadow intensity threshold will be clustered as a non-bright strut.

### False positive removal

**Fig. 5:**
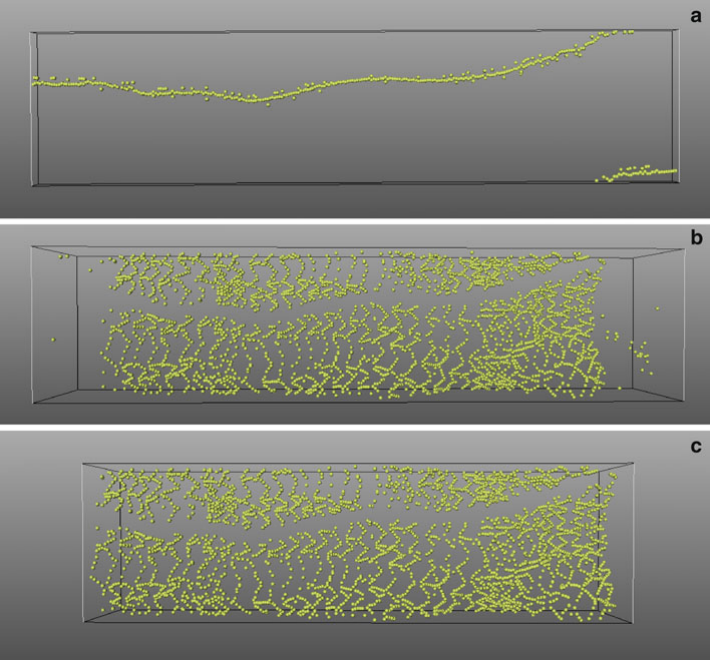
**a** shows the detected guide wire from the whole pullback run. In some frames, there is more than one guide wire because of the artifacts; **b** shows the strut results after the guide wire removal. In the beginning and the ending segments of this pullback run, no real stent exists; **c** shows the result after stented segment detection

If a guide wire is present during the image acquisition, it also reflects most of the energy and causes a trailing shadow behind it. It will be improperly recognized as a strut by our method. Compared with the real struts, a guide wire is usually located closer to the imaging catheter and its coordinates are continuous throughout the whole pullback run. Therefore, we have defined a guide wire distance threshold to measure the guide wire continuity. By using this spatial feature, a guide wire filter was developed, which searches a series of continuous candidate struts which are located closer to the imaging catheter than any other. Figure 4f shows the strut detection result after the guide wire removal. If no guide wire is used, the filter will not remove any candidate struts. Figure 5 shows the result for the guide wire removal.

In Fig. 5b, it is clearly demonstrated that there are only a few strut candidates in the proximal and distal part of the pullback run. The reason is that the pullback usually is much longer than the stent length. By analyzing the amount of struts detected in each frame of the pullback run, we can identify the stented segment automatically and remove all the strut candidates outside this segment. An example result is shown in Fig. 5c.

## Validation

To evaluate our automatic detection algorithm, we used 10 pullback runs of stented coronary segments of 7 retrospectively selected patients from a 1-year follow-up study. Eight of our polar IVOCT pullback runs have 271 frames each, and the other two pullback runs both have 541 frames.

We applied our approach to all 10 pullback runs which in total contain 3,250 frames. One observer (*A)* indicated the start point of the struts in all the images to compare them to the automated results for validation. In three pullback runs, there are a total of 19 frames that were not marked because of the very low image quality. We did not take these frames into account for our validation. In a total of 3,231 frames, 18,311 struts were marked manually.

To determine how accurate a human observer can find and indicate exactly the same location for the same strut, a second independent observer (*B*) analyzed a subset of 179 images from 8 of the 10 pullbacks. On this subset observer *A* indicated 2,033 struts, while observer *B* indicated 1,864 struts, resulting in 1,841 corresponding struts. Expert observer *A* also categorized all the IVOCT pullback runs into three groups based on the image quality: high, medium and low. This selection was based on the amount of noise in the images, and the experienced difficulty during the manual definition of the strut positions. In this paper, 4 pullback runs were assigned to the high quality group, 3 pullback runs to the medium quality group and the remaining 3 pullback runs to the low quality group. Each strut was also assigned to one of three categories based on the strut status: malapposition, apposition and covered. The malapposition group contains all the malapposed struts and the struts over the side-branches. The apposition group includes uncovered struts and the struts with minimum neointimal hyperplasia. There are 681 struts in the malapposition group, 5,382 for the apposition group and 12,248 for the covered group, respectively.

To examine the robustness of our method, we compared the results from our method with the manual results from observer *A* for different combinations of image quality and strut status. We also tested each main parameter for three values, the recommended value and ±20 % of the recommended value to investigate the sensitivity of the algorithm. In Table 3, the performance and the distance error are presented to quantify the effect of these parameter variations.

## Results

The inter-observer agreement is defined as the number of agreements divided by the average number from two observers and the agreement was found to be 95 %. The mean and standard deviation of the distance between these corresponding struts were found to be 2.9 ± 3.3 pixels. According to our experts, a 10-pixels distance (about 0.05 mm) is an acceptable distance when comparing the algorithmic results to the expert results of observer *A*. Within this acceptable distance, the mean and standard deviation of the distance error is 1.7 ± 1.1 pixels. Table 3 shows the distance error between two manual results and the distance error between the manual results of expert *A* and the automated results.

The sensitivity of the detection method is defined as the ratio between the number of struts correctly detected by our algorithm and the number of struts found by expert observer *A*. In Table 1, the average sensitivity of our automated approach for the different categories is given in percentages. The false positives (FPs) show the ratio of the number of false positives in the automatic results compared to the number of struts as defined by observer *A*. Next, the subtotals of the algorithm performance for different image qualities or different strut status categories are also given in Table 1.

**Table 1:** The sensitivity of the new algorithm for all combinations of the image quality and strut status

Strut status image quality	Malapposed (%)	Apposed (%)	Covered (%)	Subtotal (%)	FPs (%)
High	92	98	96	96	4
Medium	87	93	92	92	4
Low	92	88	89	89	6
Subtotal	91	93	94	94	4

The numbers indicate the sensitivity of the algorithm in percentages. FPs means the percentage of false positive detected struts compared to the manual results

In all groups, our method shows a good agreement with the expert results. For high, medium and low quality IVOCT images, the new method found 96, 92 and 89 % of the stent struts, respectively. For apposition, malapposition and covered status, 91, 93 and 94 % of the struts were found, respectively. The average sensitivity is 94 %. All combinations contain only a few false positives (4 %).

According to our validation, the algorithm works best for apposed struts in high quality images, since they usually appear as very clear bright spots and have nice trailing shadows. Malapposed struts may have short or blurred shadows which cause difficulties in the detection. Generally, in our low quality data set, malapposed struts are brighter than other struts. The most difficult situations are the apposed and covered struts in low quality images. Compared to the other struts, they usually have a less bright appearance and blurred shadows because of the noise or the thick coverage which causes more absorption and scattering of light. Their trailing shadows appear fuzzier and shorter compared to the other situations. Our algorithm is relatively robust in case of different image quality, but low image quality is still the main reason for false positives and false negatives. In case of severe restenosis, a strut is not visible anymore except for a weak trailing shadow. Even experts have difficulty to mark these correctly. It is also not always clear how to separate a cluster due to the structure of the stent. All these factors may affect the result of the proposed algorithm.

Eight of the pullback runs were acquired with a guide wire, while the remaining two had no guide wire. Our guide wire filter successfully detected the pullback runs that contain guide wires, and filtered all the guide wires from them. For the other two pullback runs that contain not guide wire, the filter did not remove any strut candidates.

For processing, we used a Windows XP Professional x64 Edition Version 2003 with SP2 computer with 2.0 GHz CPU and 4 GB memory. Generally, it takes less than 5 min to process a pullback run containing 271 frames with MeVisLab 2.1. We also implemented a pure C++ version of this method in QCU-CMS version 4.68 (Research version of QIvus 2.1, Medis medical imaging systems, Leiden, The Netherlands), which decreases the computing time to less than 2 min.

## Discussion

Automatic stent strut detection is important as it can simplify and speed up quantitative stent strut analysis and 3D stent reconstruction. We present a 3D detection method for stent struts in IVOCT pullback runs, which is based on the intensity features and shadow edge detection. It is also important to note that spatial information is used to remove the guide wire and the false struts in the empty frames. Because only in an IVOCT pullback run which contains a guide wire, a continuous list of strut candidates through the whole pullback run can be found. With a good performance in all situations, our method can detect stent struts robustly and independent of strut status or image quality. The validation study showed that the new method successfully detected 94 % of the 18,311 struts from 10 pullback runs. Compared to former research, our method requires no lumen contour or vessel wall segmentation and it is relatively insensitive to the image quality. Moreover, the new method does not require different modes for different strut status, so that no a priori information or user input is needed. Additionally, we presented a novel guide wire filter to classify and remove guide wire automatically.

### Parameter selection and sensitivity analysis

The whole method contains more than 10 parameters. Some parameters are related to the size of the input image, while some other parameters are fixed based on the histogram of the input image, for example, the maximal intensity threshold for trailing shadow and the slope threshold for candidate pixels detection. The most important parameters for our method are presented in Table 2. We used the same parameter rule for all the pullback runs.

**Table 2:** The major parameters used in this method

Parameter	Value	Parameter	Value
Max shadow intensity threshold	89th percentile of the histogram	Shadow edge length threshold	100 pixels
Sliding shadow size	30 pixels	Clustering distance threshold	4 pixels
Slope threshold	−48	Guide wire distance threshold	40 pixels

To evaluate our method when the parameters are changed, we varied the main parameters by ±20 %. We also computed the mean distance error between the algorithmic results and the manual results from observer A and its standard deviation. The distance error is calculated only between the successfully detected struts. The performance and the distance error are shown in Table 3 and demonstrate that even if the parameters are changed by 40 % (±20 %); the position of the struts that are detected by our algorithm does not change much.

**Table 3:** The correlation and the distance between the manual result from observer *A* and the algorithmic results with standard parameters and after the parameters are changed by ±20 %

Parameter	Performance TP [FP]* (%)			Distance Error (pixel)		
Change	0 %	−20 %	+20 %	0 %	−20 %	+20 %
Max shadow intensity threshold	94 [4]	91 [5]	94 [7]	1.7 ± 1.1	1.8 ± 1.3	1.8 ± 1.3
Sliding shadow size	94 [4]	92 [5]	93 [5]	1.7 ± 1.1	1.7 ± 1.2	1.7 ± 1.2
Slope threshold	94 [4]	93 [7]	92 [5]	1.7 ± 1.1	1.8 ± 1.3	1.8 ± 1.2
Shadow edge length threshold	94 [4]	85 [12]	88 [5]	1.7 ± 1.1	1.8 ± 1.3	1.8 ± 1.3
Clustering distance threshold	94 [4]	94 [6]	93 [4]	1.7 ± 1.1	1.8 ± 1.2	1.7 ± 1.2

The distance error and its standard deviation are measured in pixel size

* Performance TP value means the sensitivity our method. FP value in [] shows the percentage of false positive detected struts compared to the manual results

## Limitations

The presented method can cluster the strut even if only one shadow edge was detected. However, for severe in-stent restenosis, some struts are covered by such a thick layer of new tissue that only bright spots exist without any trailing shadow. The trailing edge is blurred away due to scattering in the thick layer of tissue.

**Fig. 6:**
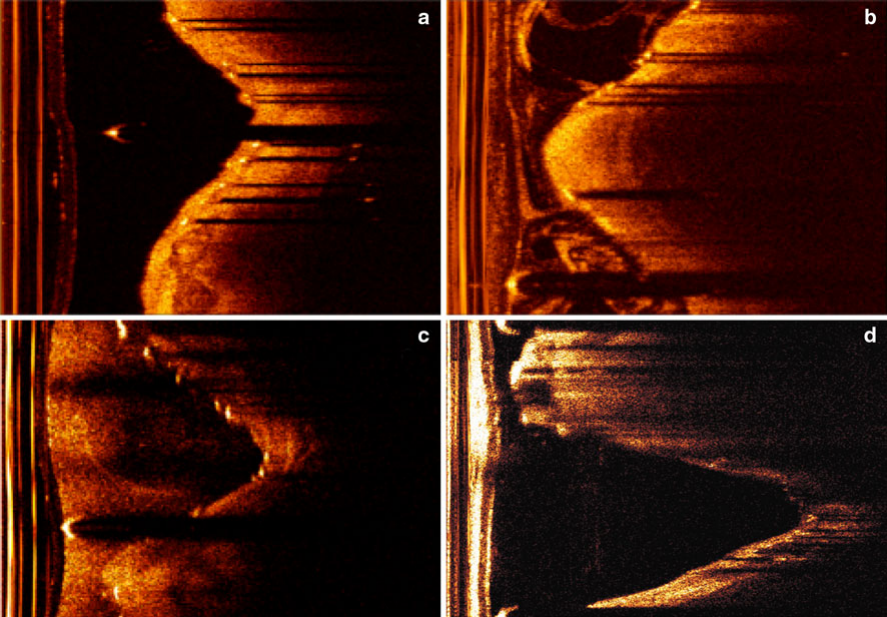
Examples of the three image quality groups; **a**, **b** show a good quality image and a medium quality image respectively. **c**, **d** are two low quality images; **c** has blurred shadows and a seaming artifact, while **d** shows some struts having only clear shadow without bright spot

In another situation, some struts have only a trailing shadow without a bright spot. These situations are very common in bad quality pullback runs as Fig. 6c, d show. Both the expert and our detection method have difficulty to deal with these cases. The edge detection has difficulties to eliminate sew-up stitches as showed in Fig. 4f. Although the shadow edge based clustering can largely eliminate this problem, false edge may introduce false struts.

Unlike the guide wire which consists of a single wire cable, the stent patterns are much more complex. Without knowing the pattern of the implanted stent, it is difficult to remove or recover stent struts within the stented segment using the spatial information. Taking into account that there are hundreds of different stent designs, using the 3D stent structure information for stent strut detection is a very challenging task. In addition, the described method is not suitable for new bioabsorbable stent struts which appear as small dark boxes instead of bright spots in IVOCT images.

## Conclusion and future research

With the high resolution of in vivo microstructure in coronary arteries, IVOCT allows a better understanding of the pathophysiology of coronary disease. We presented an automatic stent strut detection method in IVOCT image sequences regardless of strut status and image quality. The new method uses the local image intensities to detect the candidate pixels of the stent struts in preprocessed IVOCT image sequences. The edges of the trailing shadows are detected to assist the candidate pixels clustering for each strut, to reduce the false positives and to find the dark struts with clear shadows. After clustering, the guide wire is filtered out using 3D restriction. The method is independent of pre-selection the strut status or lumen/vessel wall segmentation.

A clinical data analysis was carried out to evaluate the performance of our method. Automatic results were compared with the results that were manually detected by expert observers. For IVOCT images with different quality levels, it turned out to be a robust and reliable automatic method. In conclusion, with ongoing development of IVOCT technology, our method could be helpful for stent implanting treatment evaluation, patient follow up and vascular response of different types of stent. As a next step, the result will be used as input for 3D visualization and quantification.
